# Endocytosis of the *Saccharomyces cerevisiae* Jen1 monocarboxylate-proton symporter under mildly alkaline conditions: a mechanism to prevent metabolite loss?

**DOI:** 10.1186/s12934-026-02955-6

**Published:** 2026-02-27

**Authors:** Cláudia Barata-Antunes, Marieke Warmerdam, Erik de Hulster, Inês P. Ribeiro, Clara Cardoso, Beatriz Leite, Margarida Casal, Hernâni Gerós, Jack Pronk, Joaquín Ariño, Robert Mans, Sandra Paiva

**Affiliations:** 1 Centre of Molecular and Environmental Biology, Department of Biology, University of Minho, 4710-057 Braga, Portugal; 2 Institute of Science and Innovation for Bio-Sustainability (IB-S), University of Minho, 4710-057 Braga, Portugal; 3 Department of Biotechnology, Delft University of Technology, Van der Maasweg 9, 2629 HZ Delft, The Netherlands; 4 Life and Health Sciences Research Institute (ICVS), University of Minho, Braga, Portugal; 5 Centre for the Research and Technology of Agro-Environmental and Biological Sciences, University of Trás-os-Montes and Alto Douro, Vila Real, Portugal; 6 Centre of Biological Engineering (CEB), Department of Biological Engineering, University of Minho, Braga, Portugal; 7 Institut de Biotecnologia i Biomedicina & Departament de Bioquímica i Biologia Molecular, Universitat Autònoma de Barcelona, Cerdanyola del Vallès, Spain

**Keywords:** Transporters, Carboxylic acids, Endocytosis, Alkali stress, Lactate, pH, continuous cultivation.

## Abstract

**Background:**

The *Saccharomyces cerevisiae* Jen1 transporter mediates electroneutral proton symport of lactate and pyruvate. In lactate-grown batch cultures, growth-coupled medium alkalinization was previously shown to coincide with endocytosis of Jen1.

**Results:**

To investigate the physiological relevance of pH-dependent Jen1 endocytosis, *S. cerevisiae* was grown in carbon-limited continuous cultures on a mixed ethanol-lactate feed. When applying a linearly increasing pH (6.75–7.25) to these cultures, lactate and pyruvate concentrations in the external medium progressively increased. Up to a culture pH of 7.0, these extracellular concentrations aligned with a predicted thermodynamic equilibrium of reversible, Jen1-mediated electroneutral carboxylate-proton symport. Consistent with earlier reports, pronounced Jen1 internalization occurred above pH 7.0. At these mildly alkaline pH values, a more pronounced increase of residual lactate concentrations and transcriptional upregulation of genes involved in oxidative phosphorylation were consistent with increased cellular energy demands.

**Conclusion:**

This study reveals how pH-dependent regulation of carboxylate transporters shapes cellular adaptation to changing environmental conditions. Insights into these regulatory mechanisms can inform strategies to optimize microbial cell factories operating under variable pH regimes in industrial settings. The integrated analysis of transport, Jen1 localization, and transcriptional responses in growing continuous cultures uncovered physiological challenges associated with electroneutral carboxylate/proton symport under mildly alkaline conditions. The data support the hypothesis that Jen1 internalization evolved to prevent intracellular metabolite loss under unfavorable pH conditions.

**Supplementary Information:**

The online version contains supplementary material available at 10.1186/s12934-026-02955-6.

## Background

In nature as well as in industrial contexts, the yeast *Saccharomyces cerevisiae* faces changes in its environment. Cells can adapt to these changes, which include fluctuations in nutrient availability and extracellular pH, by reprogramming their metabolism, for instance by inducing or repressing transcription of specific genes [1–4]. Adaptation can also occur post-translationally, for example by promoting internalization (endocytosis) and degradation of plasma membrane (PM) transporters that are deleterious or dispensable under specific conditions [5–7].

Jen1 is an *S. cerevisiae* transmembrane transporter protein that mediates electroneutral symport of monocarboxylate anions such as lactate and pyruvate with a proton [8–11]. It is expressed and localized at the PM during growth on non-fermentable carbon sources, including lactate, pyruvate, acetate and ethanol [12]. A variety of signals induce Jen1 internalization and degradation, including exposure to glucose [10, 13–19], rapamycin, or cycloheximide ([20], see [5] for a review). When yeast cells are growing on monocarboxylates, the resulting alkalinization of the extracellular medium coincides with Jen1 endocytosis and degradation [20].

In shake-flask cultures, concentrations of biomass, substrate, oxygen and protons all change during growth. These dynamics can affect gene expression [21] and other cellular processes, including endocytosis of proteins localized at the PM. This complexity makes it difficult to unequivocally assess the impact of extracellular pH on Jen1-mediated carboxylate transport and Jen1 endocytosis. In continuous cultures, key culture parameters such as specific growth rate, temperature, biomass concentration, pH, and oxygen availability can be controlled independently and maintained at desired setpoints [22] (Fig. S1). Use of continuous cultures thereby facilitates identification of the impact of individual culture parameters (e.g., extracellular pH) on microbial physiology and gene expression [23].

The PM ATPase (Pma1) of *S. cerevisiae* cells generates an electrochemical proton gradient, the proton motive force (PMF), across the PM by pumping out a proton at the expense of ATP [24]. The PMF consists of the pH gradient (∆pH = pH_in_ - pH_out_) and the electrical potential difference resulting from the charge gradient, generated by the movement of positive or negative charges to the extracellular space (∆*ψ* = *ψ*_in_ – *ψ*_out_, in V) [25]. Because Jen1 mediates electroneutral monocarboxylate/proton symport, with a stoichiometry of 1 H^+^ (proton): 1 monocarboxylate (anion) [10], only the pH gradient (∆pH) contributes to the driving force for accumulative monocarboxylate uptake [25]. Monocarboxylate transport by Jen1 is reversible and has also been implicated in monocarboxylate export [26–29].

**Fig. 1:**
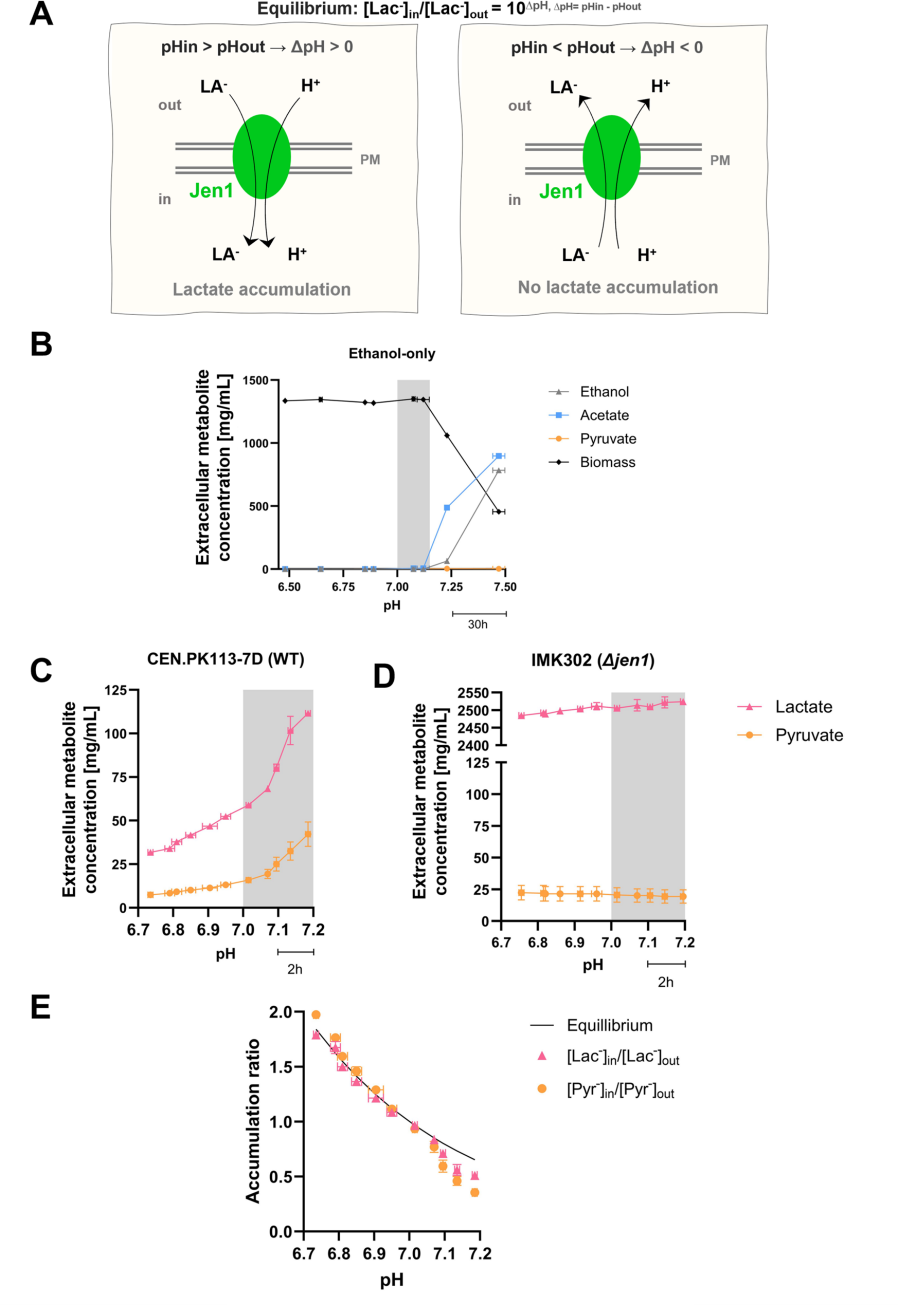
Physiology of *S. cerevisiae* strains in aerobic, carbon-limited continuous cultures subjected to a linear increase of extracellular pH. Continuous cultures grown at a dilution rate of 0.07 h^− 1^ were continuously fed with SM containing 50 mM ethanol, either with or without 33 mM L-lactate. Continuous-culture experiments (panels B-E) were performed as independent duplicates and the data are presented as the mean, with error bars representing the standard error of the mean (SEM). **A** Schematic representation of the reversible ΔpH-dependent symport of lactate anions (La^−^) and protons (H^+^) by Jen1. At extracellular pH values below the cytosolic pH (ΔpH > 0), equilibrium of Jen1-mediated transport involves intracellular lactate accumulation, while the reverse situation occurs when ΔpH < 0. **B** Concentrations of biomass, ethanol and pyruvate in a continuous culture of *S. cerevisiae* CEN.PK113-7D grown on ethanol as sole carbon source and subjected to a linearly increasing pH (0.0083 ≈ 0.01 pH unit h^− 1^). The time scale bar (30 h) represents the duration required for each 0.25 pH unit increase. **C** Concentrations of lactate and pyruvate in a continuous culture of *S. cerevisiae* CEN.PK113-7D, fed with a mixture of ethanol and L-lactate and subjected to a linearly increasing pH (0.05 pH unit h^− 1^). The time scale bar (2 h) represents the duration required for each 0.1 pH unit increase **D** Concentrations of lactate and pyruvate in a continuous culture of a congenic *Δjen1 S. cerevisiae* strain, fed with a mixture of ethanol and lactic acid and subjected to a linearly increasing pH (0.05 pH unit h^− 1^). **E** Accumulation ratios (AR, internal carboxylate concentration divided by external carboxylate concentration) of lactate and pyruvate during the pH ramp experiments shown in panel C and the corresponding independent replicate experiments. The solid black line indicates the AR calculated from the equilibrium equation (AR = 10^(pHin – pHout)^) and a pH_in_ of 7.0. AR values were calculated from extracellular concentrations based on the assumption that intracellular concentrations of lactate and pyruvate concentrations were constant and independent of extracellular pH. (**B**/**C**/**D**) Metabolite concentrations represent residual steady-state concentrations in the culture

Facilitation of bidirectional transport implies that, when the external pH exceeds the internal pH, equilibrium of Jen1-mediated transport will occur at external substrate concentrations that are higher than those in the cytosol. We hypothesise that under these conditions, removal of Jen1 from the PM limits or prevents cellular ‘leakage’ of monocarboxylates that are transported by Jen1 (Fig. 1A). To test this hypothesis and to further explore pH-dependent Jen1 endocytosis, *S. cerevisiae* was first grown in aerobic, carbon-limited continuous cultures on a mixture of ethanol and lactate. These continuous cultures were then subjected to a linear increase of the external pH, starting below and ending above pH 7.0, which, based on literature, is close to the cytosolic pH of this yeast over a range of extracellular pH values [30–33]. Using this experimental set-up, we measured extracellular concentrations of the Jen1-transported metabolites lactate and pyruvate, as well as the subcellular localization of Jen1. To further investigate cellular responses of lactate-consuming cultures at supra-optimal pH values, transcriptome analysis was performed on steady-state continuous cultures grown at selected pH values.

## Materials and methods

### Yeast strains and storage conditions

The *S. cerevisiae* strains used in this study are listed in Table 1. For prolonged storage, the strains were grown at 30 °C, 200 rpm, in YPD (1% (w/v) yeast extract, 1% (w/v) peptone and 2% (w/v) glucose) medium. After overnight growth, glycerol was added to a final concentration of 30% (v/v) and 1 mL aliquots were stored at – 80 °C.

**Table 1:** List of the strains used in this work

Strain	Genotype	Reference/source
Ace55	*W303-1 A JEN1::GFP::KanMX leu2 ura3 trp1 his3 ade2*	Our laboratory collection
CEN.PK113-7D	MATa MAL2-8c SUC2	[34]
CB191	CEN.PK113-7D *JEN1::GFP::KanMX*	This work
CB270	CEN.PK113-7D *JEN1::GFP:: HghMX*	This work
IMK302	CEN.PK113-7D *jen1::loxP-KanMX4-loxP*	[35]

### Strain construction

All the *S. cerevisiae* strains generated in this work were constructed as follows: firstly, DNA fragments were amplified by PCR (Accuzyme DNA Polymerase, Bioline, or Supreme NZYProof DNA Polymerase, Nzytech) with specific oligonucleotides (listed in Table 2) using yeast genomic DNA or plasmid DNA. The resulting PCR products were introduced in *S. cerevisiae* cells, by following the polyethylene glycol (PEG)/lithium acetate (LiAc) protocol [36], and the resulting transformants were selected on geneticin (G418 disulfate, Alfa Aesar; J62671) or hygromycin B (Alfa Aesar; J60681) according to the resistance (R) marker present (geneticin for *kan*R and hygromycin for *hgh*R). Strain construction was carried out such that the number of cell divisions, which could lead to additional unintended mutations, was kept to a minimum.

Specifically, the strain CB191 (Table 1) was obtained as following: the DNA cassette *JEN1::GFP::kanMX* was PCR amplified from the genomic DNA of the strain Ace55 (Table 1) with the primers W303fw and W303rev. The DNA cassette was then introduced in the CENPK113-7D strain and positive transformants were confirmed by colony PCR using the primers K3 fw and W303rev or W303fw and K2 rev.

The CB270 strain (Table 1) was obtained by replacing the kanamycin resistance (*kanR) sequence* by the hygromycin resistance (*hghR)* DNA region in the CB191 strain. In detail, the *hghR* DNA sequence was first amplified from pAG32 plasmid [37] using primers 150_MX4fwd and 149_MX4rev (Table 2), which align 50 bps before the end of TEF promoter and 50 bps after the beginning of TEF terminator, respectively. Since KanR sequence is also flanked by TEF promoter and terminator regions, the 50 bp homology arms facilitate homologous recombination, allowing the replacement of *kanR* with *hghR*. Positive transformants were confirmed by colony PCR using primers 150_MX4fwd and 324_Hygrev.

**Table 2:** List of the primers used in this work

Primer name	Sequence (5´→ 3´)
W303fw	GATTTGTCCTCTCCTGTTATGAAG
W303rev	ATCTTGCTAGTCTTAACGGCTGTTA
K3 fw	CCATCCTATGGAACTGCCTC
K2 rev	CGATAGATTGTCGCACCTG
150_MX4fwd	AAAATCTTGCTAGGATACAGTTCTC
149_MX4rev	ACAAATGACAAGTTCTTGAAAACAA
324_Hygrev	TGCATCATCGAAATTGCCGTCAACCAA

### Media and cultivation conditions

For the ‘quick’ and ethanol-only pH ramp experiments, *S. cerevisiae* pre-cultures were grown aerobically at 30 °C, 200 rpm, in 500 mL Erlenmeyer flasks containing 100 mL of synthetic medium (SM) [38] supplemented with 7.5 g/L of ethanol. Ethanol and filter-sterilised vitamins were added after autoclaving the other constituents of SM. For all other batch cultivations, 20 g/L of glucose was used instead of ethanol.

Continuous cultures were carried out in 2 L laboratory bioreactors (Getinge Applikon, Delft, The Netherlands) with a 1 L working volume. Cultures were grown aerobically at 30 °C in synthetic medium (for more details see [38, 39] and Table S1) with 33 mM (≈ 2.9 gL^− 1^) L-lactic acid and 50 mM (≈ 2.3 gL^− 1^) ethanol as sole carbon-limited sources. Ethanol and filter-sterilized vitamins (Table S1 and [38]) were added after autoclaving the medium. After the batch phase, the medium pumps were switched on, resulting in the continuous addition of 70 mL/h of SM, supplemented with 33 mM L-lactic acid and 50 mM ethanol, to the culture. The working volume (1.0 L) was kept constant using an effluent pump controlled by an electric level sensor, resulting in a dilution rate (D) of 0.07 h^− 1^. The culture was aerated with 500 mL/min air and stirred at 800 rpm with Rushton impellers and pH was maintained at 6.0, 6.5, 7.0, 7.1 or 7.2 by automated addition of 2 M KOH or 2 M H_2_SO_4_ (Fig. S1). Continuous cultures were considered to be in steady-state (SS) when, after at least 5 volume changes, the culture dry weight, extracellular metabolite concentrations, the CO_2_ production and O_2_ consumption rates did not differ more than 5% over 2 volume changes. At steady state, chemostat-grown cells are maintained in balanced exponential growth at a fixed specific growth rate dictated by the dilution rate [22, 40]. The continuous-culture runs were limited to fewer than 15–20 volume changes after inoculation, to avoid evolutionary adaptation of the cultures.

For the pH ramp experiments, steady-state cultures were first established at pH 6.5 and subsequently a pH ramp from pH 6.5 to pH 7.5 was programmed in which the medium pH was automatically increased over the course of 120 h (ethanol-only control) or 100 h (Jen1-GFP, optical density was automatically monitored over the course of this experiment). For the ‘quick’ pH ramp experiments comparing WT to the otherwise congenic *Δjen1* strain, steady-state was established at pH 6.75, after which the pH ramp program increased the reactor pH setpoint 6.75 to pH setpoint 7.25 with 0.05 units per hour.

The off-gas signal (CO_2_ concentration) was automatically monitored over the course of all bioreactor experiments. Samples for dry weight, HPLC (for external metabolites analysis) and fluorescence microscopy (to follow the cellular localization of Jen1-GFP) were taken when the culture reached specific extracellular pH values (as indicated in the figure legends).

The cultures were independently performed at least in duplicate (*n* ≥ 2).

### Analytical methods

#### Optical density

Optical density at 660 nm was manually measured using a Jenway 7200 spectrophotometer (Bibby Scientific, Staffordshire, UK).

#### Off-gas

Off-gas was first cooled in a condenser at 2 °C and then dried with a Perma Pure Dryer (Permapure, Toms River, NJ). Subsequently, CO_2_ and O_2_ concentrations were measured using an NGA 2000 Rosemount gas analyser (Emerson, St Louis, MO, USA).

#### Dry weight

Culture dry weights were determined by filtrating 10 mL of culture using dry-, pre-weighed nitrocellulose filters with a pore size of 0.45 μm (Gelman Laboratory, Ann Arbor, MI, USA). The filters were washed twice with demineralized water before and after the filtration of the culture samples. After filtration, the filters were dried in a microwave for 20 min at 360 W and weighed again. Final dry weights are the average of duplicates performed for each continuous culture.

#### High performance liquid chromatography (HPLC)

The extracellular metabolites and/or substrates present in the culture supernatants and media were analyzed by HPLC using an Agilent 1260 HPLC, equipped with a Bio-Rad HPX 87 H ion-exchange column, operated at 60 °C with 5 mM H_2_SO_4_ as mobile phase at a flow rate of 0.600 mL∙min^− 1^. For sampling steady state cultures, ≈ 5 mL of culture broth from the bioreactor was rapidly sampled into a syringe containing pre-cooled stainless steel balls (4 mm, Fabory Laman Zoetermeer). The supernatant was immediately separated from the cells by filtration through a 0.45-µm pore size filter (PVD membrane, Merck Millipore Ltd.) (adapted from [41]).

#### Epifluorescence microscopy and quantitative analysis

For the detection of Jen1-GFP cellular localization, a volume of 1 mL culture was collected and concentrated by a factor of 100 by centrifugation (5000 rpm, 1 min). 4 µL of each sample was then directly and immediately visualized, without fixation, on a Zeiss Axio Imager Z1 microscope (Carl Zeiss AG, Oberkochen, Germany) with appropriate filters using a 100x magnification objective. Cell dry weight of all samples was measured prior to microscopy analysis and is reported in (Table S6).

The quantitative analysis of the signal at PM/Total signal was performed using ImageJ software (version 1.53k) as described in [17, 19]. Specifically, two ellipses were drawn for each cell (*n* ≥ 300) using the “magic wand” tool: the first ellipse was drawn around the entire cell (total fluorescence), and the second ellipse was drawn inside the cell, to exclude the plasma-membrane localized signal. The integrated density (IntDens) of both of these regions of interest was measured. The difference between the IntDens of the 1st ellipse (total cell) and the second ellipse (inside cell) gives the signal at the plasma membrane (signal at PM = IntDens[1st ellipse]−IntDens[2nd ellipse]). Data were represented as the ratio between the signal at PM and the total signal. An ordinary one-way ANOVA analysis was used followed by a Tukey´s multiple comparisons test using Prism 9.0 (GraphPad software, version 9.2.0). Because fluorescence ratios were compared across more than two independent experimental groups (different extracellular pH conditions), one-way ANOVA was selected as the appropriate test to assess overall differences among means. When significant, Tukey’s test was applied to correct for multiple pairwise comparisons. The *P* values are indicated (NS: *P* > 0.05; *, *P* < 0.05; **, *P* < 0.01; ***, *P* < 0.001; ****, *P* < 0.0001).

### RNA extraction, sequencing and analysis

#### Culture sample collection

Steady state culture samples (± 240 mg of cells) were collected directly from the bioreactor and instantaneously frozen in liquid nitrogen and stored at − 80 °C for further RNA sample extraction.

#### RNA extraction

RNA extraction was performed using an acid phenol-chloroform protocol adapted from established yeast RNA isolation methods [42]. This extraction procedure efficiently separates RNA into the aqueous phase while retaining most DNA in the organic phase, thereby minimizing DNA contamination. Specifically, 800 µL of “crushed ice” bits, from the − 80 °C samples, were transferred to Eppendorf tubes and thawed on ice. Eppendorfs were then centrifuged at 8000 *g*, 4 min, 0 °C and the pellet was resuspended in 200 µL cold RNase-free water. Subsequently, 400 µL of acid phenol-chloroform was quickly added and the mixture was centrifuged at 8000 *g*, for 4 min, at room temperature. The aqueous layer was transferred to a new tube and 400 µL of chloroform was added. The mixture was centrifuged again at 8000 *g*, 4 min, room temperature, and the upper layer was transferred to a new tube. 20 µL of 3 M Na-acetate and 500 µL of 100% (v/v) ethanol (− 20 °C) were added. After mix by vortexing, the samples were incubated at − 20 °C for at least 15 min. The samples were then centrifuged for 15 min, 4 °C, at 8000 *g* and the pellet was washed with 500 µL 80% (v/v) ethanol (− 20 °C). The samples were vortexed, centrifuged again and, after removal of all the ethanol, the pellet was resuspended in 20 µL RNase-free water. The samples were kept at room temperature for at least 2 h to dissolve the RNA. The concentration, purity and quality of the total RNA samples were also determined using Tapestation (Agilent Technologies 2200) and NanoDrop 2000 (Thermo scientific).

#### RNA sequencing

RNA samples [8 samples, four different pHs (6.0; 6.5; 7.0 and 7.1) in duplicate] were sent to Novogene (UK) for mRNA sequencing (RNA-seq). Non-directional libraries were prepared and checked with Qubit and real-time PCR for quantification and bioanalyzer for size distribution detection. Quantified libraries were pooled and sequenced on an Illumina platform.

#### Analysis of RNA-Seq data

Raw reads (fastq format) were processed through FASTQ software [43] to yield clean data by removing low quality reads and reads containing adapters. The quality of the data was examined with the FastQC software (https://www.bioinformatics.babraham.ac.uk/projects/fastqc/). The average quality per read (Phred score) was above 35 for all samples. Mapping of paired-end clean reads (2 × 150 nt) were aligned to the *S. cerevisiae* CEN.PK113-7D genome (assembly ASM26988v1, obtained from https://fungi.ensembl.org/) with the Hisat2 v2.0.5 software [44]. Percentages of overall alignment were between 92 and 98%. The featureCounts (v1.5.0-p3) program was used to count the number of reads mapped to each gene. Differential expression analysis was conducted using DESeq2 R package (1.20.0) using the Benjamini and Hochberg’s approach to adjust the resulting *p*-values [45]. Genes with a *p*-value < = 0.01 found by DESeq2 and a log_2_ fold change of +/- 0.6 were assigned as differentially expressed. Gene Ontology analyses for functional profiling were done with the g: Profiler server (https://biit.cs.ut.ee/gprofiler/gost) using *S. cerevisiae* S288c strain data. The statistical domain scope used was “only annotated genes” and the g: SCS algorithm was applied for significance threshold (set at a *p*-value of 0.05). The search for transcription factors binding sites in the promoters of the selected genes was carried out at the Regulatory Sequence Analysis Tools (RSAT) server (https://rsat.france-bioinformatique.fr/fungi/) with the matrix-scan tool [46] and selecting an upstream segment of 800 nt (allowing overlapping with ORF). The transcription factor matrix data from the JASPAR database [47] was used and a *p*-value of 10^− 4^ was set as significance threshold. Four individual sets of 174 promoters randomly extracted were also analysed and used as reference.

## Results

Extracellular lactate and pyruvate concentrations in carbon-limited continuous cultures subjected to a linear pH ramp.

A mixed-substrate continuous cultivation system was implemented to study the impact of extracellular pH on activity and subcellular localization of the Jen1 monocarboxylate-proton symporter. Lactate was fed to these cultures along with ethanol, which is transported across the yeast PM by passive diffusion (Fig. S2) and neither represses *JEN1* promoter activity [12] nor interferes with Jen1 internalization and degradation. This combination was carefully selected based on prior findings showing that vacuolar sorting of Jen1 requires the presence of its substrate (lactate or pyruvate). In batch cultures, medium alkalinization alone—without lactate (e.g., during growth on glycerol)—did not lead to complete removal of Jen1 from the plasma membrane [20]. Thus, lactate appears to be essential to activate the internalization mechanism under alkaline pH. However, in chemostat cultures, continuous growth must be maintained to avoid washout. Since Jen1 becomes degraded at higher pH, cells progressively lose the ability to import lactate as a sole carbon source. Ethanol was therefore included to support growth when lactate uptake was compromised, enabling the culture to remain at steady state without introducing regulatory interference in Jen1 expression or trafficking.

To investigate up to which external pH value *S. cerevisiae* CEN.PK113-7D can grow on ethanol at a dilution rate of 0.07 h^− 1^, continuous cultures grown on ethanol as sole carbon substrate were subjected to a pre-programmed pH ramp. When the culture pH was linearly increased from 6.50 to 7.50 at a rate of 0.01 pH units h^− 1^, incomplete ethanol utilization occurred above pH 7.20 (Fig. 1B and Table S2). This observation and a progressive decrease of the biomass concentration in the cultures indicated that, above pH 7.20, the maximum specific growth rate on ethanol declined to below the dilution rate of 0.07 h^− 1^.

Similar carbon-limited continuous cultures of *S. cerevisiae* CEN.PK113-7D fed with a mixture of 33 mmol L^− 1^ L-lactate and 50 mmol L^− 1^ ethanol (dilution rate 0.07 h^− 1^) were first allowed to reach steady state at pH 6.75. Subsequently, a pre-programmed pH ramp was initiated, during which the culture pH was linearly increased from pH 6.75 to 7.20 at a rate of 0.05 pH units h^− 1^. During this pH ramp, extracellular concentrations of the Jen1 substrates lactate increased with increasing pH. This culture pH-dependent increase of the extracellular lactate concentration became more pronounced above pH 7.0 (Fig. 1C and Table S3). An additional pH-ramp experiment (pH 6.75 to pH 7.25, 0.01 pH unit h^− 1^) was performed with a congenic *Δjen1* strain. The growth rates of this strain and the reference were assessed in batch cultures grown on ethanol, revealing identical rates, which suggests that there were no unintended differences in metabolic activity between the strains (Fig. S3). The high residual lactate concentrations in these cultures (Fig. 1D and Table S4) corresponded with the lactate concentration in the medium feed. This observation confirmed early work showing that, in wild-type *S. cerevisiae*, Jen1 is exclusively responsible for lactate uptake [9].

In the ethanol-lactate pH-ramp experiment with the *S. cerevisiae JEN1* strain CEN.PK113-7D, not only the extracellular concentrations of lactate, but also those of pyruvate increased with increasing pH (Fig. 1C and Table S3). These results indicated that, as the extracellular pH increased, higher extracellular lactate concentrations were needed to sustain a specific growth rate of 0.07 h^− 1^. A simultaneous increase of the extracellular concentrations of pyruvate is consistent with its Jen1-mediated export. To interpret these observations, we consider the impact of extracellular pH on reversible carboxylate-proton symport.

When extracellular and intracellular pH are the same (∆pH = 0), Jen1-mediated electroneutral proton symport reaches equilibrium at identical intra- and extracellular concentrations of its carboxylate substrates. Based on multiple studies in which intracellular pH in *S. cerevisiae* was found to be close to 7.0 at extracellular pH values ranging from 6.0 to 7.5 [30–33, 48] we assumed this situation to occur at an extracellular pH of 7.0. This yielded estimated cytosolic concentrations of lactate and pyruvate of 0.62 and 0.18 mM, respectively. The estimated intracellular concentrations are close to reported K_m_ values of purified *S. cerevisiae* L-lactate dehydrogenase for lactate (0.5 mM [49]), and of isolated *S. cerevisiae* mitochondria for pyruvate (0.3 mM [50]) .

We hypothesise that, at the fixed specific growth rate in the continuous cultures, intracellular concentrations of lactate and pyruvate only depended on the (fixed) specific growth rate and were independent of external pH. At extracellular pH values of 6.75–7.0, accumulation ratios of lactate and pyruvate, calculated based on these assumptions from measured extracellular concentrations, closely agreed with the theoretical equilibrium (internal/external concentration = 10^∆pH^ when pK_a_ < < pH_out_, pH_in_; Fig. 1E). As anticipated, calculated accumulation ratios for lactate and pyruvate decreased below 1 at pH values above 7.0. However, at those pH values, accumulation ratios were even lower than predicted based on the abovementioned assumptions. This observation may reflect that, at external pH values above 7.0, higher intracellular concentrations of pyruvate and lactate are needed to increase in vivo activity of lactate dehydrogenase and mitochondrial pyruvate uptake and, thereby, enable the generation of additional ATP for countering alkaline stress.

### Localization and internalization of Jen1 in response to a linear increase of extracellular pH

**Fig. 2:**
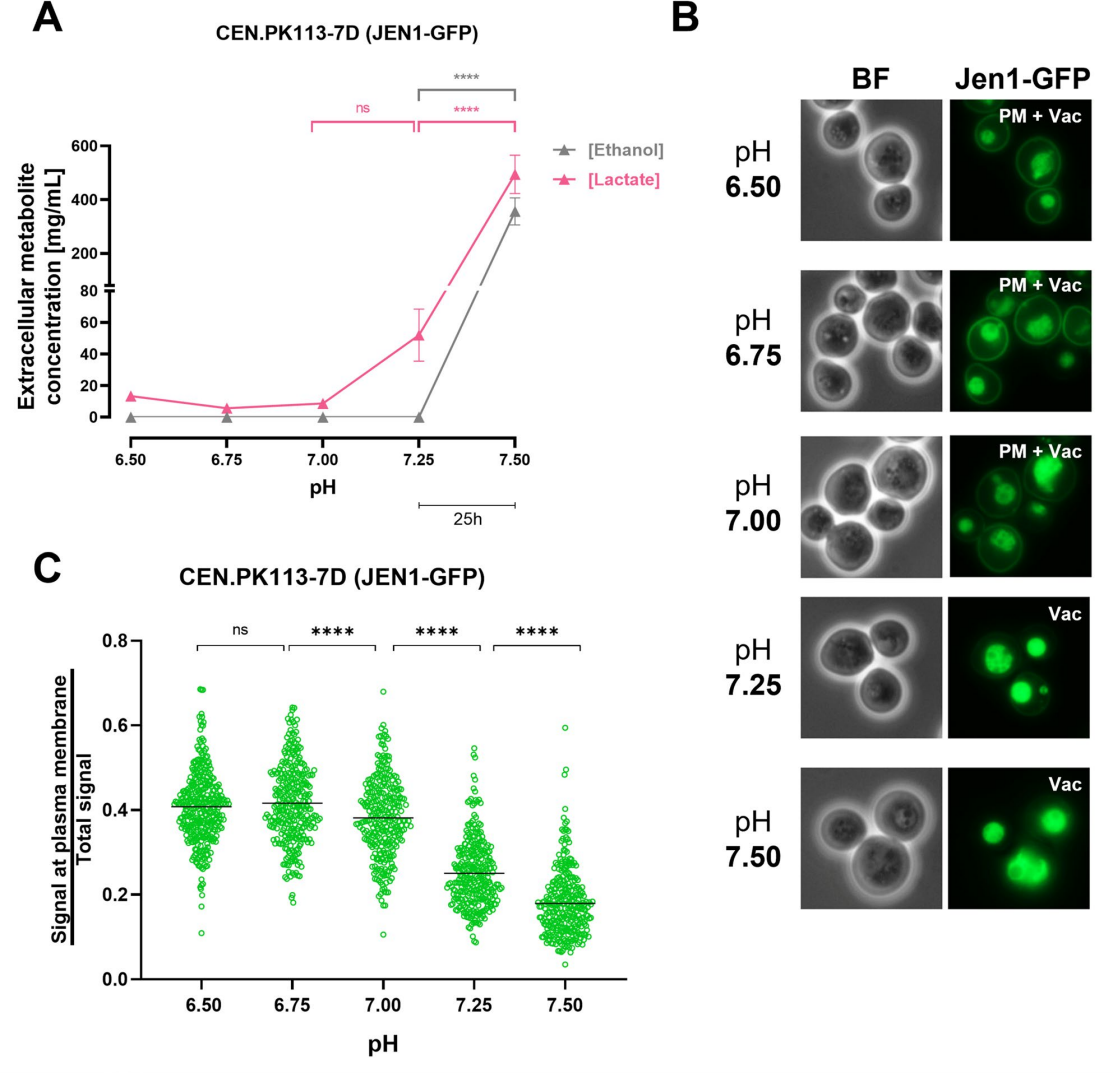
Evaluation of Jen1-GFP localization in *S. cerevisiae* CB270 (*JEN1::GFP::HghMX*) grown in aerobic, carbon-limited continuous cultures subjected to a linear increase of extracellular pH. Duplicate continuous cultures grown at a dilution rate of 0.07 h^− 1^ were continuously fed with SM containing 50 mM ethanol and 30 mM L-lactate and subjected to a linearly increasing pH ramp (0.01 pH units h^− 1^). **A** Extracellular concentrations of ethanol and lactate (mg L^− 1^) represent residual steady-state concentrations in the culture. Data are presented as the means of two individual experiments, with error bars representing the standard error of the mean (SEM). ns, not significant (*P* = 0.8095); ****P < 0.0001 (two-way ANOVA analysis with a Tukey’s multiple comparisons test). The time scale bar (25 h) represents the duration required for each 0.25 pH unit increase. **B** Visualization of Jen1-GFP by epifluorescence microscopy at the indicated extracellular culture pH. The primary localization of the Jen1-GFP is described at the top right corner of each fluorescence microscopy image. PM, plasma membrane; Vac, vacuole; BF, bright field. **C** Quantitative analysis of the ratio of peripheral fluorescence over total fluorescence. Data are represented as scatter plot (*n* ≥ 300 cells for each plot), median values are indicated by horizontal lines. ns, not significant (*P* > 0.05); ****P < 0.0001 (ordinary one-way ANOVA analysis with Tukey’s multiple comparisons test)

In contrast to lactate, pyruvate was not included in the feed of the continuous cultures. The observed extracellular pyruvate concentrations in the pH-ramp experiments therefore reflected net export of pyruvate, a known substrate of Jen1 [9, 51], from the cells (Fig. 1C). Preventing release of pyruvate, a central intermediate in yeast metabolism [52], might provide a rationale for the previously reported removal of Jen1 from the PM upon exposure to alkaline pH in shake-flask cultures [20]. To specifically investigate the influence of extracellular pH on subcellular localization of Jen1, a pH-ramp experiment (pH 6.5–7.5, 0.01 pH unit h^− 1^) was performed with continuous cultures of a congenic *S. cerevisiae* strain CB270 that constitutively expresses a Jen1-GFP fusion protein (Fig. 2).

Culture samples were collected at every 0.25 pH interval for lactate and acetate measurements, as well as microscopy analysis. The residual concentration of lactate in continuous cultures did not significantly change between pH 6.5 and 7.0 (Fig. 2A and Table S5). However, above pH 7.0, the lactate concentration steeply increased from 0.009 ± 0.001 g/L (mean ± s.d.) to 0.495 ± 0.101 g/L at pH 7.5. No extracellular ethanol was detected when cultures were grown at pH values between 6.5 and 7.25, but at pH 7.5 an extracellular concentration of 0.36 ± 0.07 g L^− 1^ ethanol was measured, coincident with a decrease in biomass dry weight (Table S6). This observation is consistent with results from cultures grown on ethanol as sole carbon source (Fig. 1B), in which cells were unable to keep up with the dilution rate of the continuous cultures (0.07 h^− 1^) at pH values above 7.25.

Fluorescence microscopy (Fig. 2B) clearly revealed presence of Jen1-GFP at the PM at culture pH values of 6.5, 6.75 and 7.0. In contrast, at pH 7.25 and 7.50, visual inspection no longer revealed fluorescence at the PM and, instead, showed a predominant localization of fluorescence in the vacuole (Fig. 2B). In line with these visual observations, the ratio of peripheral fluorescence over total fluorescence by image quantitative analysis showed a decrease from 0.42 ± 0.091 (mean ± s.d.) at pH 6.75 to 0.18 ± 0.077 at pH 7.5 (Fig. 2C). These results demonstrate that the threshold extracellular pH at which Jen1-GFP is removed from the PM lies between pH 7.0 and 7.25.

### Extracellular metabolite concentrations and Jen1 internalization in steady-state cultures grown at different extracellular pH values

Dynamic pH-ramp experiments do not necessarily reflect the physiology of cells that are fully adapted to a specific extracellular pH. Therefore, based on results from the pH-ramp experiments, duplicate steady-state continuous cultures (dilution rate, 0.07 h^− 1^) of the Jen1-GFP expressing strain CB270 were grown on ethanol/lactate at four different culture pH values: pH 6.0, pH 6.5 (Jen1 at PM in pH ramp experiments), pH 7.0 and pH 7.1 (close to Jen1 internalization threshold pH identified in pH-ramp experiments). Upon reaching steady state, culture samples were taken for metabolite measurements, microscopy and transcriptome analysis.

Attempts to establish stable steady-state cultures at pH 7.2, where Jen1 was largely removed from the PM in the pH-ramp experiments, were unsuccessful due to culture wash-out. This indicated that, at this pH, cells were unable to sustain a specific growth rate that matched the dilution rate (0.07 h^− 1^). This result was consistent with results from pH-ramp experiments with ethanol-grown cultures of strain CEN.PK113-7D (Fig. 1B), in which pH 7.2 coincided with the point at which extracellular ethanol and acetate started to appear.

**Fig. 3:**
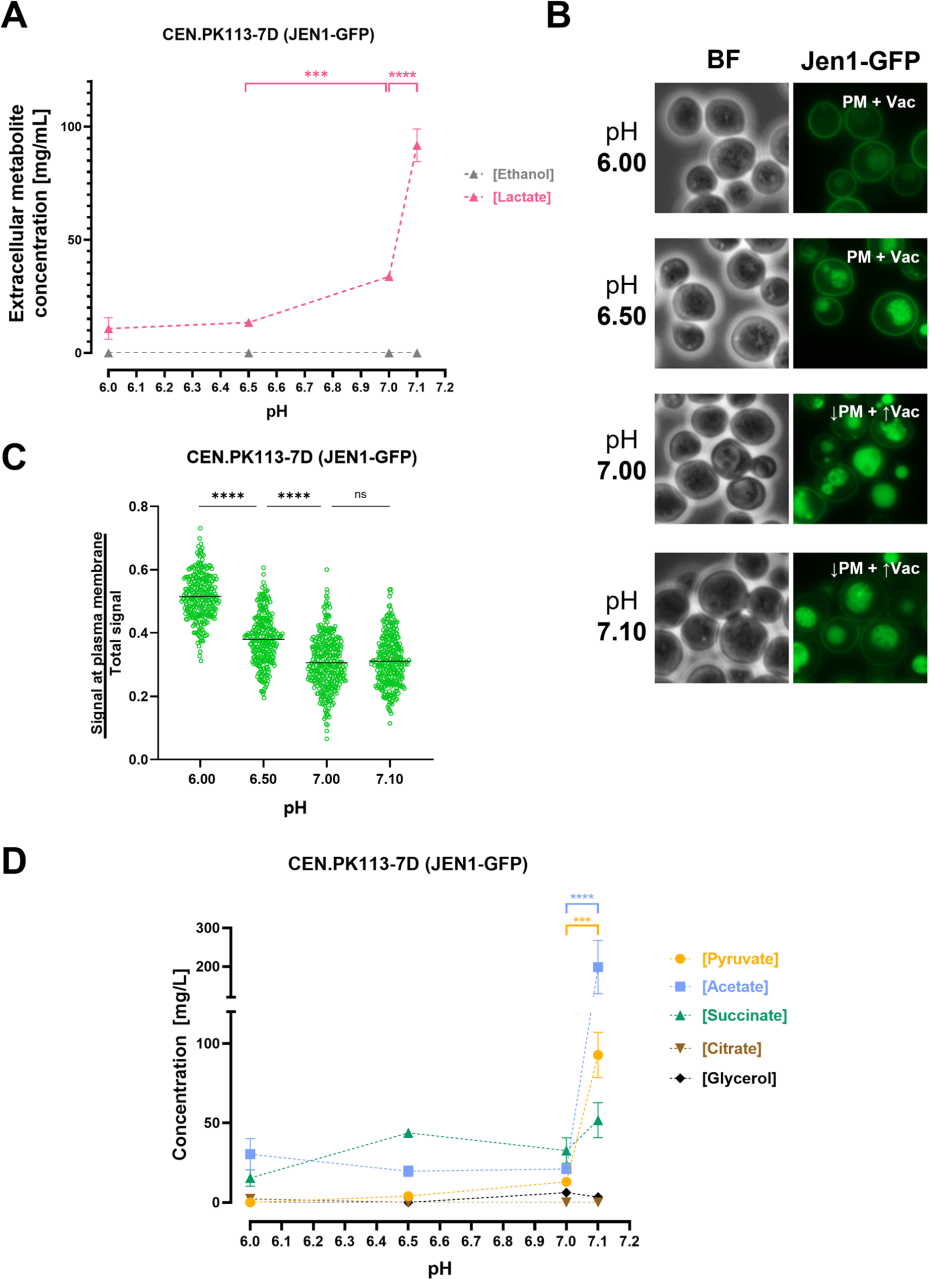
Extracellular metabolite concentrations and Jen1 internalization in steady-state continuous cultures grown at different extracellular pH values. Steady-state, carbon-limited continuous cultures of *S. cerevisiae* CB270 (CEN.PK113-7D *JEN1::GFP::HghMX*) grown at a dilution rate of 0.07 h^− 1^ were fed with SM containing 50 mM ethanol and 30 mM L-lactate. Culture pH was maintained at 6.0, 6.5, 7.0, 7.1 or 7.2 by automated addition of 2 M KOH or 2 M H_2_SO_4_ (see Material and Methods). **A** Extracellular concentrations of ethanol and lactate (mg L^− 1^) represent residual steady-state concentrations in the culture. Data are presented as the means of at least two individual experiments, with error bars representing the standard error of the mean (SEM), ***P = 0.0004; ****P < 0.0001 (two-way ANOVA analysis with Tukey’s multiple comparisons test). **B** Visualisation of Jen1-GFP by epifluorescence microscopy at the indicated extracellular culture pH. The primary localization of Jen1-GFP is at the top right corner of each fluorescence microscopy image. PM, plasma membrane; Vac, vacuole; BF, bright field. **C** Quantitative analysis of the ratio of peripheral fluorescence over total fluorescence. Data are represented as scatter plot (*n* ≥ 270 cells for each plot), median values are indicated by horizontal lines. ns, not significant (*P* > 0.05); ****P < 0.0001 (ordinary one-way ANOVA analysis with Tukey’s multiple comparisons test. **D** Extracellular concentrations of metabolites other than lactate and ethanol (mg L^− 1^) represent residual steady-state concentrations in the culture. Data are presented as the means of at least two individual experiments, with error bars representing the standard error of the mean (SEM), ***P = 0.0006; ****P < 0.0001 (two-way ANOVA analysis was performed, followed by a Tukey’s multiple comparisons test)

At culture pH up to 7.1, no extracellular ethanol was detected, indicating that the steady-state cultures were able to maintain a specific growth rate of 0.07 h^− 1^. The residual concentration of lactate in the cultures, which was approximately 13 mg L^− 1^ at pH 6.0 and 6.5, and 30 mg L^− 1^ at pH 7.0, increased to 92 mg L^− 1^ at pH 7.1 (Fig. 3A; see also Table S7).

Fluorescence microscopy of cells from steady-state cultures grown at pH 6.0 and 6.5 showed localization of Jen1-GFP at the PM and in the vacuole (Fig. 3B). In cultures grown at extracellular pH values of 7.0 and 7.1, less Jen1-GFP was found at the PM, while the fluorescence in the vacuole increased. In line with these observations, image analysis yielded a peripheral/total fluorescence ratio of 0.51 ± 0.07 at an extracellular pH of 6.0 and of approximately 0.3 at extracellular pH values of 7.0 and 7.1 (Fig. 3C).

In addition to the extracellular concentrations of lactate, also those of pyruvate, acetate, succinate, citrate, and glycerol were determined in culture supernatants of each steady-state culture (Fig. 3D, Table S8). These analyses indicated a 7- to 9- fold difference in the extracellular concentrations of pyruvate and acetate between cultures grown at pH 7.0 and pH 7.1. No such pronounced differences were observed for the other metabolites.

### Transcriptome sequencing (RNA-seq) analysis of steady state-, aerobic-, continuous cultures of *S. cerevisiae* grown at different extracellular pH values

**Fig. 4:**
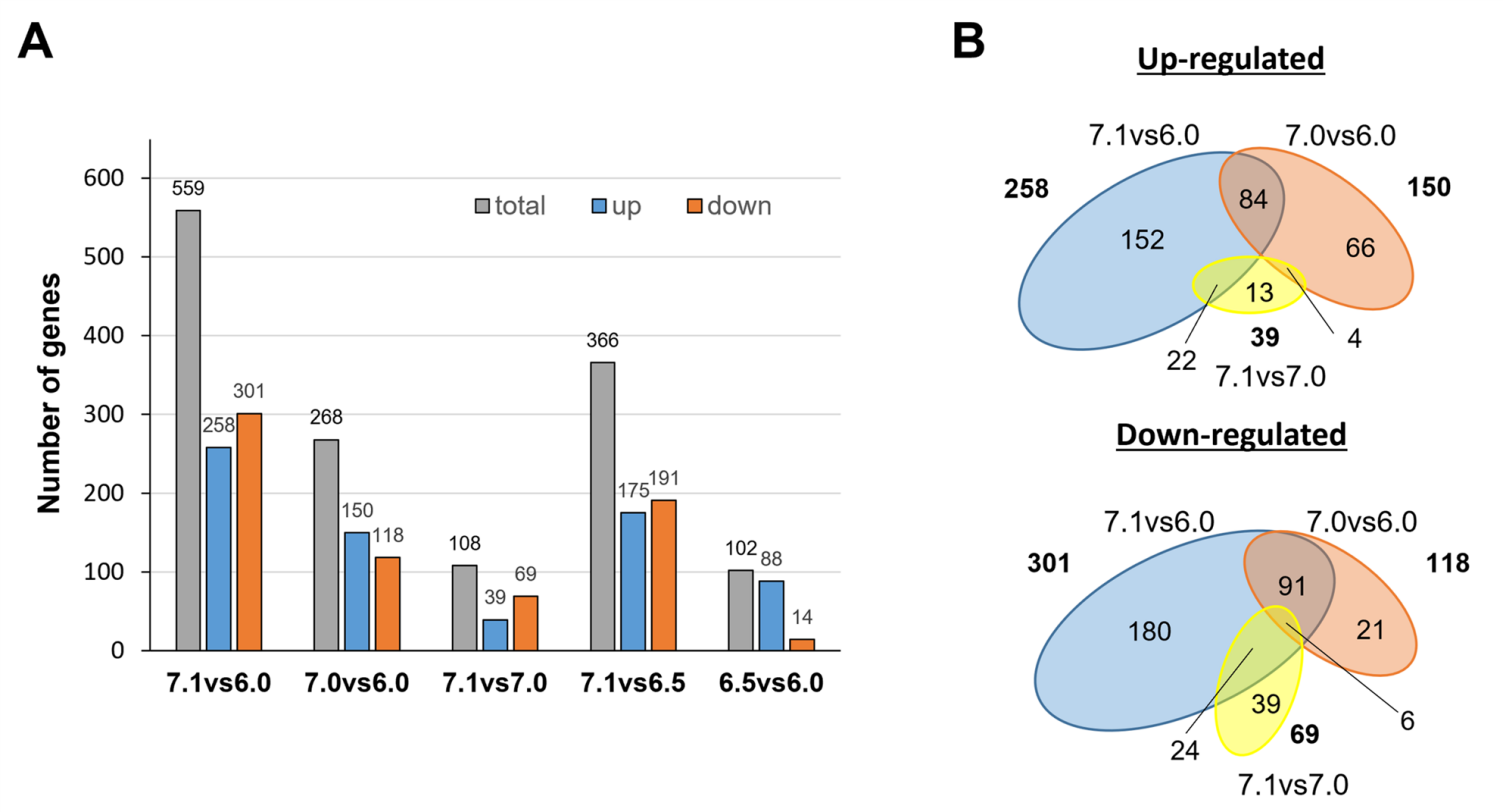
Transcriptional impact of external pH in steady-state continuous cultures of the *S. cerevisiae* strain CB270. Continuous cultures were grown at a dilution rate of 0.07 h^− 1^ and fed with SM containing 50 mM ethanol and 30 mM L-lactic acid at pH 6.0, pH 6.5, pH 7.0 and pH 7.1. **A** Pairwise comparison of transcriptome data from cultures grown at different pH values (6.5vs6.0; 7.0vs6.0; 7.1vs6.0; 7.1vs6.5 and 7.1vs7.0). The grey column represents the total number of altered genes, and the blue and orange columns represent the number of up-regulated or down-regulated genes, respectively. **B** Venn diagrams of differentially expressed genes (DEG) for the pH 7.1vs6.0 (blue), 7.0vs6.0 (orange), and 7.1vs7.0 (yellow) data sets. The number of genes in each dataset are denoted in bold and is proportional to the area of the ellipses. The overlapping region indicates the numbers of DEGs that are common in the compared groups

After defining the pH range at which Jen1 localized to the vacuoles, we aimed to investigate the metabolic status of the cell at different pHs and identify the pathways potentially involved in Jen1 internalization. To achieve this, samples of steady-state, aerobic continuous cultures of *S. cerevisiae* grown at pH extracellular values of pH 6.0, 6.5, 7.0, and 7.1 were collected for RNA sequencing. Because all RNA-seq samples were obtained from steady-state continuous cultures operated at identical dilution rate, biomass concentration, and nutrient conditions, differences in gene expression therefore reflect solely the effect of extracellular pH and not differences in growth phase, nutrient availability, or overall physiological state. The RNA-seq analysis dataset was combined, and comparisons were made between the datasets obtained at the different pH values. Because the differences in pH of the investigated cultures were small, a relatively low-stringency threshold (log_2_ >0.6 or <-0.6, with p-value < 0.01) was applied to select for differentially expressed genes (DEGs). As expected, the effect on the number of DEGs was related to the intensity of the pH stress. Thus, the increase of pH from 6.0 to 6.5 affected a relatively small number of genes, barely above 100 (most of them induced, Figs. 4 and S4). A further increase of the culture pH to 7.0 resulted in a higher number of genes with altered expression levels, with a particular increase in the number of those whose mRNA decreased. Interestingly, a relatively small increase of the culture pH by 0.1 units (7.1vs6.0 compared with 7.0vs6.0) doubled the number of mRNAs that were altered, with a nearly 3-fold increase in the number of repressed genes. Comparison of the data obtained at pH 7.0 with that found at pH 7.1 (Fig. 4A) yielded 108 genes with altered expression (mostly repressed). Considering that this change in pH results in a relatively minor alteration in proton concentration in comparison with the other changes examined, this transcriptional response can be considered meaningful.

**Fig. 5:**
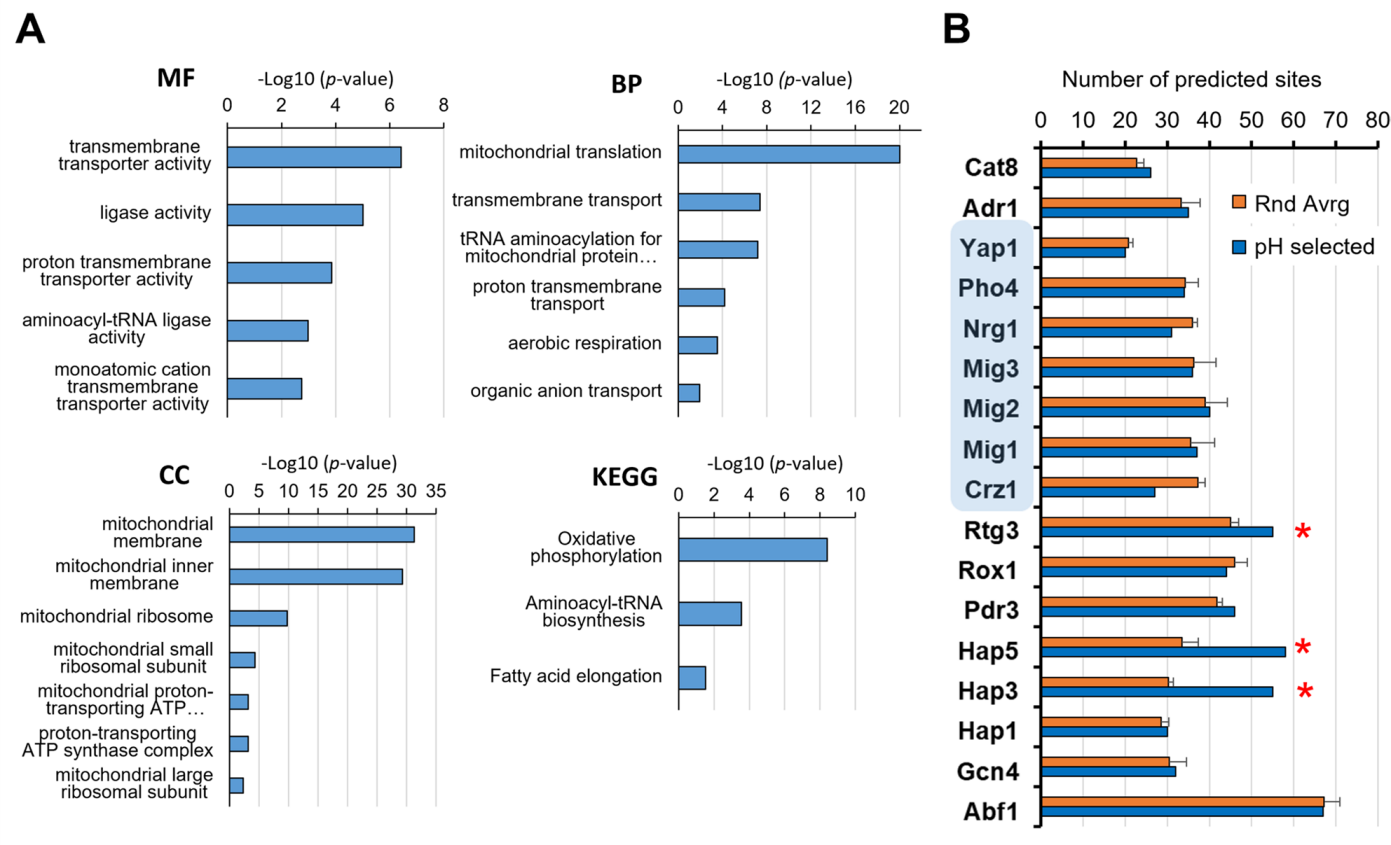
Gene Ontology and promoter analysis of the 174 genes specifically induced at the pH 6.0 to 7.1 transition. **A** The GO terms of the MF (Molecular Function), BP (Biological Process), and CC (Cellular component) domains enrichment of the 174 differentially expressed (up-regulated) genes. GO, Gene Ontology; KEGG, Kyoto Encyclopaedia of Genes and Genomes biological pathways database. The *p*-value threshold was set at 0.05. **B** The JASPAR database matrices were used to scan for transcription factor binding sites in the promoters of the mentioned genes (pH selected). As reference, four sets of 174 randomly selected genes were also scanned and the mean ± SEM of the number of predicted sites is offered for comparison. The asterisks denote binding sites whose abundance is markedly increased in the pH selected gene cohort. The blue shading denotes transcriptions factors whose activity is affected by alkalinization according to the literature (see main text)

The transition from pH 6.0 to pH 7.0 or 7.1 resulted in changes consistent with previously reported data from cells subjected to mild alkalinization (see Discussion). Thus, diverse genes related to iron uptake and metabolism (*SIT1*, *FIT2*, *FIT3*, *FRE1*, *FET3*, and *HXM1*), high-affinity phosphate (*PHO84*, *PHO89*), or hexose transport (*HXT14*, *HXT10*, *HXT2*) were induced by the shift to either pH 7.0 or 7.1 (Fig. S6). Figure 4B shows the overlap of up-regulated or down-regulated genes between the following pH comparisons 7.1vs6.0, 7.0vs6.0 and 7.0vs7.1. It is worth noting that 174 of the 258 genes (59.7%) induced when comparing pH 7.1 and 6.0 were not found to be induced when comparing pH 7.0 and 6.0, whereas 204 out of 301 (67.8%) were found repressed in the former case but not in the latter. This suggests that the transition from pH 7.0 to pH 7.1, although it might appear quantitatively minor, has a notable impact in the expression profile of the cells. Since the most pronounced internalization of Jen1 seemed to be triggered by events during this transition, we found it pertinent to further examine the 174 genes specifically induced in cells exposed to the pH change from 6.0 to 7.1, but not from 6.0 to 7.0. As shown in Figs. 5A and S6A, this set of 174 induced genes was strongly enriched for genes related to mitochondrially-related events, with 70 genes (40%) encoding mitochondrial membrane-associated proteins (Table S9). Thirty-three genes were directly related to mitochondrial translation, including those encoding ribosomal proteins (such as *MRPL17*, *MRPL3*, *MRPL10*, and others), translation factors (such as *MRF1*, *MEF1*, *IFM1*, or *TUF1*), or RNA aminoacyl ligases (including *SLM5*, *MSR1*, *MST1*, *ISM1* and others). It is worth noting that genes encoding cytosolic ribosomal genes were not induced at all (Figs. 5A and S6A). In addition, sixteen genes were related to oxidative phosphorylation, either as members of the electron transport chain (such as *RIP1*, *SDH3*, *COX6*, COX*10*, *COX11* and others) or as components of the ATP synthase complex (*ATP2*, *ATP4*, *ATP5*, *ATP16*, *ATP17*, and *TIM11*). Additionally, substantial downregulation of mitochondrially-related genes is observed in the shift from pH 6.0 to 7.0, but not when pH changes from 6.0 to 7.1 (Fig. S6B).

The promoter region of the 174 genes was investigated for predicted transcription factor binding sites. As shown in Fig. 5B, such promoter regions were not enriched for consensus sequences corresponding to activators or repressors (shaded in blue) that, according to the literature [53–55], are known to play a relevant role in the overall adaptation to alkalinization. In fact, a decrease for the calcium-calcineurin activated Crz1 transcription factor was observed. In contrast, an enrichment for Rtg3 and, in particular, for Hap3 and Hap5 was detected. Rtg3 is a positive regulator of the retrograde pathway, which responds to mitochondrial dysfunction by adapting cell metabolism to the loss of tricarboxylic acid (TCA) cycle activity ([56, 57], reviewed in [58]). Hap3 and Hap5 are subunits of the Hap2p/3p/4p/5p CCAAT-binding complex, a transcriptional activator of respiratory gene expression (TCA cycle and oxidative phosphorylation genes) [59, 60]. Interestingly, the minimal change from pH 7.0 to 7.1 resulted in the transcriptional upregulation of 39 genes, of which only four were also induced in the transition from pH 6.0 to 7.0. Notably, these were all genes encoding amino acid permeases: *DIP5*, which transports mainly dicarboxylic amino acids, but also Gln, Asn, Ser, Ala, and Gly; *BAP3*, a permease involved in uptake of cysteine and branched chain amino acids; *GNP1*, a broad specificity amino acid permease; and *TAT1*, which transports branched chain amino acids, as well as Tyr, Trp and His. Gene Ontology analysis of the 35 non-induced genes yielded a clear enrichment (*p*-value = 1.8E-6) in genes encoding components of the mitochondrial inner membrane thus reinforcing the notion that the transition from pH 7.0 to 7.1 has a considerable impact on the regulation of mitochondrial function.

## Discussion

Electroneutral symport of a proton and a monovalent carboxylate anion across the yeast plasma membrane, as mediated by Jen1, allows for ΔpH-driven accumulative anion import when the external pH is lower than the intracellular pH. For example, at an external pH of 5, which is near the optimum pH for growth of *S. cerevisiae* [61] and at a cytosolic pH of 7, equilibrium of Jen1-mediated lactate transport occurs at an intracellular-to-extracellular accumulation ratio of 100 of the anion. When corrected for the pK_a_ of lactic acid of 3.86 (see [62] for equation), this corresponds to a combined lactate/lactic acid accumulation ratio of 93-fold. However, as the extracellular pH increases, the contribution of ΔpH to the driving force for lactate uptake decreases. When the external pH becomes higher than the cytosolic pH, this contribution even becomes negative. The results presented in this study provide new insights into the physiological consequences of the mode of energy coupling of Jen1-mediated monocarboxylate transport and into the physiological relevance of Jen1 endocytosis in situations where the extracellular pH equals or exceeds the cytosolic pH.

At a constant cytosolic pH, the equilibrium of Jen1-mediated transport ([lactate]_in_/[lactate]_out_ = 10^ΔpH^) dictates that, as the external pH increases, maintenance of a constant intracellular lactate concentration requires the extracellular lactate concentration to increase. Such an increase of the extracellular lactate concentration was indeed observed in continuous cultures grown on a lactate-ethanol mixture that were subjected to a linearly increasing extracellular pH (Fig. 1). Moreover, extracellular lactate concentrations measured in those cultures were consistent with Jen1-mediated transport operating at or close to thermodynamic equilibrium. This implies that, under the experimental conditions, in vivo activity of Jen1 did not constrain lactate transport across the PM. In natural environments with near-neutral or mildly alkaline conditions, the associated higher extracellular lactate concentrations are likely to negatively affect competition for lactate with microorganisms that employ Δψ- or ATP-coupled transporters and, consequently, can achieve much higher accumulation ratios. Based on our experiments with the Jen1 model system, we therefore hypothesize that electroneutral anion-proton symport is likely to predominantly occur in microorganisms adapted to environments with a pH below 7.

The thermodynamic constraints of electroneutral H⁺/monocarboxylate symport are expected to apply broadly, but the physiological consequences at near-neutral or mildly alkaline pH are likely to depend on transporter diversity and cellular bioenergetics. Yeasts adapted to neutral/alkaline niches may mitigate net carboxylate loss through multiple strategies, including (i) expression of multiple JEN-family permeases with distinct substrate specificities or transport properties, enabling a shift in transporter usage as ΔpH decreases, (ii) robust cytosolic pH homeostasis and/or rapid metabolic consumption of imported carboxylates that limits cytosolic accumulation, and (iii) greater reliance on transport modes that incorporate Δψ or ATP as an additional energy source. At present, pH-dependent endocytosis of Jen-family monocarboxylate transporters has primarily been documented for *S. cerevisiae* Jen1, and it remains to be tested whether similar regulation occurs in yeasts that frequently encounter neutral or mildly alkaline environments. Comparative experiments applying identical growth conditions and monitoring transporter localization, extracellular carboxylate accumulation, and intracellular pH would therefore help clarify the extent to which Jen1 endocytosis represents a conserved response versus a species-specific adaptation. Moreover, while the present work explores the shift from acidic to neutral pH, future studies could explore the reversibility of transporter endocytosis in a shift to acidic pH.

In the pH-ramp experiments shown in Fig. 1, calculated accumulation ratios of pyruvate, for which Jen1 is the only known efficient transporter in wild-type *S. cerevisiae* [51], at different external pH values, were virtually identical to those of lactate (Fig. 1E). Equilibration of in- and extracellular concentrations of essential intracellular metabolites such as pyruvate can potentially have a large impact on microbial cells, as illustrated by a simple example. One gram of yeast biomass has an intracellular volume of approximately 2 mL [63]. In a culture containing 100 mg yeast biomass L^− 1^, intracellular volume therefore accounts for only 0.02% of the total culture volume. Assuming an intracellular pyruvate concentration of 1 mmol L^− 1^, equilibrium of Jen1-mediated transport at ΔpH = 0 will result in a situation where the biomass contains 0.2 µmol pyruvate, while the external medium contains 1 mmol pyruvate (with a mass of 88 mg, equal to 88% of the total cell mass in the culture). In other words, when placed in medium without pyruvate, the cells would export their total weight in the form of pyruvate before equilibrium of transport over the membrane is reached. As illustrated in Figs. 1 and 3, the extent of this metabolite loss will become even more pronounced at pH values above 7. The metabolic burden of symporter-mediated electroneutral carboxylate efflux can be even further enhanced when, in natural environments, biomass concentrations are lower, or when competing microorganisms and/or dilution continuously decrease extracellular concentrations of the ‘leaked’ carboxylate. Although *S. cerevisiae* is clearly not optimally adapted for growth at neutral or mildly alkaline environments, this does not rule out an evolutionary significance of preventing metabolite leakage during transient exposure to such conditions. Use of continuous cultures, in which specific growth rate and other growth parameters were carefully controlled, enabled unequivocal identification of extracellular pH as a key trigger for removal of Jen1 from the plasma membrane. The maximum culture pH of approximately 7.2, imposed by the continuous cultivation set-up, did not allow us to capture the complete internalization of Jen1 that was previously observed in batch cultures [20], which demonstrated complete Jen1 internalization. However, in cultures grown at pH 7.0 and 7.1, the majority of a Jen1-GPF fusion protein was found in the vacuole. This result supports the hypothesis that Jen1 internalization has evolved as a mechanism to prevent intracellular metabolite loss during exposure to alkaline environments.

**Fig. 6:**
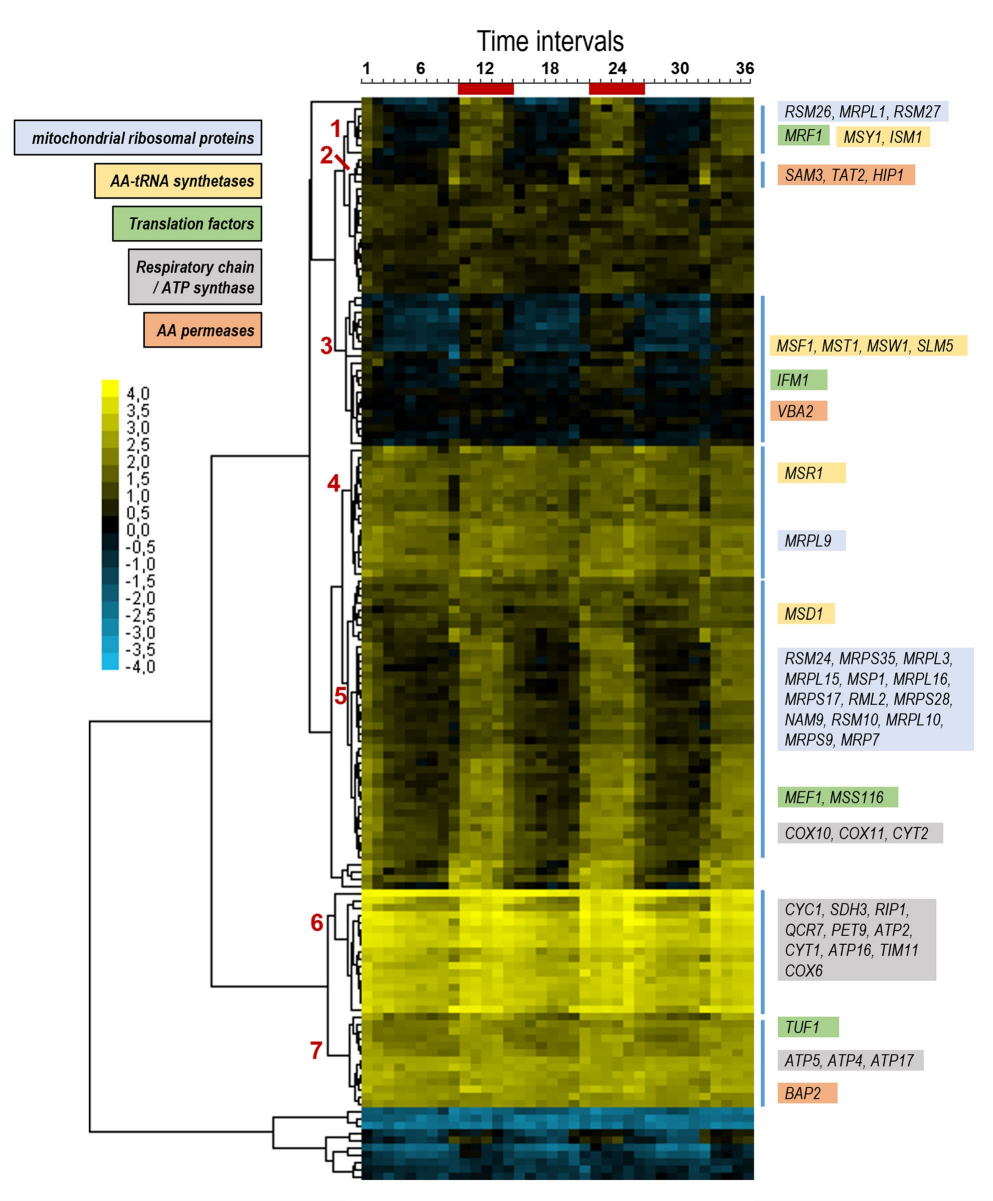
Periodic profile of genes specifically induced at the pH 6.0–7.1 transition. Our list of 174 genes was crossed with the expression data provided by Tu and coworkers [66] which described the cyclic expression profile of yeast cells grown under continuous, nutrient limiting conditions. The data for the 149 genes common to both datasets was clustered with the Cluster 3.0 software (Euclidean distance, average linkage) [71] and plotted with JavaTreeView [72]. The numbers on the left identify the main clusters, with relevant genes shown on the right according the indicated functional colour code. Time intervals at the top correspond to the interval of sampling (approximately 25 min). The red bars show the time intervals corresponding to the reductive/building (R/B) period (see main text and reference [66])

Transcriptome data corresponding to the transitions from pH 6.0 to pH 7.0 or 7.1 reveals an increase in mRNA levels of genes involved in iron and copper uptake and metabolism, as well as in high-affinity phosphate and sugar transport. These changes fit well with previously reported data that different research groups obtained from exponentially growing batch cultures that were subjected to moderate alkalinization [53, 64, 65]. However, our data also show that the transition to pH 7.1 triggers a specific response that is not observed in cells shifted from pH 6.0 to 7.0. This response involves induction of genes involved in oxidative phosphorylation (Fig. 6) and suggests that growth at pH values above 7.0 requires increased rates of respiration-coupled ATP generation. At those pH values, extracellular concentrations of lactate and pyruvate in pH-ramp experiments could no longer be fitted based on an assumed strict homeostasis of intracellular lactate and pyruvate concentrations (Fig. 1). In line with the transcriptome data, the higher inferred intracellular lactate and pyruvate concentrations in cultures grown at pH values above 7.0 (Fig. 1E) may reflect increased lactate dissimilation enabled by elevated intracellular substrate concentrations of key enzymes and metabolites in the dissimilatory pathway. Our previous study demonstrated that Jen1 internalization, triggered by the prolonged growth on lactate and consequent alkalinization, seemed to involve the Bul1 α-arrestin and the TORC1 pathway [20]. However, the transcriptomic data obtained from continuous cultures in this study did not reveal an upregulation of the TORC1 pathway when pH 6.0 to 7.1 conditions were compared (Fig. 5). The differences in the background strains and in growth conditions between these studies, where the continuous cultures in this work were exposed for longer periods to the alkaline conditions, could have led to the use of an alternative intracellular pathway for Jen1 degradation.

Tu et al. [66] showed that *S. cerevisiae* cells grown under continuous, nutrient-limited conditions, after a short initial starvation period, exhibit highly periodic cycles related to respiratory bursts. They identified three repetitive superclusters, oxidative (Ox), reductive/building (R/B), and reductive/charging (R/C), thus defining three major phases of the yeast metabolic cycle. We soon realized that the top 40 R/B genes identified, showing stronger periodicity, included 13 of our 174 genes, whereas none of them appeared in the top 40 Ox or R/C lists. To better quantify this apparently specific correspondence, we performed a cluster analysis with the transcriptomic data reported in [66] for the 149 genes in common with our 174 gene list. As presented in Fig. 6, with few exceptions all these genes are expressed following the R/B supercluster pattern. As the mentioned R/B supercluster peaks when cells are about to cease oxygen consumption, it could be interpreted as a preparatory step to meet an acute requirement to increase respiratory capacity. It must be noted that the expression of many of the genes identified in the specific switch from pH 6.0–7.1 does not seem linked to a general response to alkalinization, as the promoters of these genes are not enriched in binding sites for any of the diverse transcription factors known to shape the alkaline pH response [55]. Instead, such response is clearly connected to the mitochondria and appear to indicate a first step in intensifying the capacity for mitochondrial respiration. This is reflected in the enrichment in Rtg3 and, specially, Hap3 and Hap5 binding sites in their promoters (Fig. 5B), as these transcription factors are pivotal components of the adaptation to respiration [60]. Interestingly, an increase in the TCA cycle activity and respiratory rate when batch cultures of yeast cells were switched to pH 7.5 was reported [67].

## Conclusion

Extracellular metabolite analyses, subcellular localization studies and transcriptome profiling of controlled continuous cultures subjected to defined pH changes enabled a deeper understanding of the mode of action, regulation and thermodynamic constraints of carboxylate transport by Jen1 at neutral and alkaline pH.

These findings have practical implications for the design and operation of microbial cell factories in dynamic industrial environments, where pH fluctuations can negatively affect growth and productivity. Notably, H⁺-coupled carboxylate uptake becomes less favourable at elevated pH, and leakage of carboxylic intermediates from the use of substrates such as ethanol may contribute to growth challenges. Managing pH and transporter regulation could thus enhance process robustness. Furthermore, pH-dependent regulation of carboxylate transporters is likely to be relevant for production and/or consumption of industrially relevant carboxylic acids other than lactate and pyruvate, such as formate, acetate and 3-hydroxypropionate. Interest is growing in using CO_2_ derived substrates such as acetic and formic acid. Understanding carboxylate transporter dynamics under different pH conditions can support the selection and development of yeast strains tailored for such bioconversion processes and thereby ultimately advance sustainable biotechnologies, including agro-food bioprocesses.

While the thermodynamic constraints identified here are expected to apply broadly to electroneutral H⁺/carboxylate symport, their physiological consequences may differ across species depending on transporter diversity, regulation, and cellular pH homeostasis. It will therefore be interesting to extend the same approach to investigate pH-dependent activity and localization of additional H^+^/carboxylate symporters in *S. cerevisiae* and other eukaryotes. Promising targets include the *S. cerevisiae* H^+^-uracil symporter Fur4, which has been implicated in the loss of intracellular uracil under alkaline conditions [68], as well as acetate transporters. The latter are of particular interest because acetate is released by both ethanol-grown (Fig. 1B) and glucose-grown [39] *S. cerevisiae* cultures at extracellular pH values above 7, and because different energy-coupling mechanisms have been proposed for its transport (reviewed in [69, 70]).

Taken together, these observations point to a broader role for transporter energetics in shaping cellular physiology across pH regimes. The impact of transporter energetics on fitness has been extensively studied in cultures grown at low pH (e.g. weak acid uncoupling, substrate-accelerated death related to proton symporters, and ATP driven exporters such as PDR12). This work highlights that transporter energetics can also affect metabolite retention and cellular fitness at supra-optimal pH values and thereby affect microbial adaptation to natural and industrial environments.

## Supplementary Information

Below is the link to the electronic supplementary material.

Supplementary Material 1.

Supplementary Material 2.

## Data Availability

RNA-Seq data was deposited at *Repositório de Dados da Universidade do Minho* (dataRepositóriUM) under study https://doi.org/10.34622/datarepositorium/LWNKLQ.
